# Potential Short-Term Memory Induction as a Promising Method for Increasing Drought Tolerance in Sweetpotato Crop Wild Relatives [*Ipomoea* series *Batatas* (Choisy) D. F. Austin]

**DOI:** 10.3389/fpls.2020.567507

**Published:** 2020-09-03

**Authors:** Fernando Guerrero-Zurita, David A. Ramírez, Javier Rinza, Johan Ninanya, Raúl Blas, Bettina Heider

**Affiliations:** ^1^ Genetics, Genomics and Crop Improvement Division, International Potato Center, Lima, Peru; ^2^ Latin American & Caribbean Regional Program, International Potato Center, Lima, Peru; ^3^ Crop and Systems Science Division, International Potato Center, Lima, Peru; ^4^ Crop Husbandry Department, Universidad Nacional Agraria La Molina, Lima, Peru

**Keywords:** senescence delay, foliar area, leaf temperature, ^13^C discrimination, drought stress memory, Batatas complex

## Abstract

Crop wild relatives of sweetpotato [*Ipomoea* series *Batatas* (Choisy) D. F. Austin] are a group of species with potential for use in crop improvement programs seeking to breed for drought tolerance. Stress memory in this group could enhance these species’ physiological response to drought, though no studies have yet been conducted in this area. In this pot experiment, drought tolerance, determined using secondary traits, was tested in 59 sweetpotato crop wild relative accessions using potential short-term memory induction. For this purpose, accessions were subjected to two treatments, i) non-priming: full irrigation (up to field capacity, 0.32 w/w) from transplanting to harvest and ii) priming: full irrigation from transplanting to flowering onset (FO) followed by a priming process from FO to harvest. The priming process consisted of three water restriction periods of increasing length (8, 11, and 14 days) followed each by a recovery period of 14 days with full irrigation. Potential stress memory induction was calculated for each accession based on ecophysiological indicators such as senescence, foliar area, leaf-minus-air temperature, and leaf ^13^C discrimination. Based on total biomass production, resilience and production capacity were calculated per accession to evaluate drought tolerance. Increase in foliar area, efficient leaf thermoregulation, improvement of leaf photosynthetic performance, and delayed senescence were identified in 23.7, 28.8, 50.8, and 81.4% of the total number of accessions, respectively. It was observed that under a severe drought scenario, a resilient response included more long-lived green leaf area while a productive response was related to optimized leaf thermoregulation and gas exchange. Our preliminary results suggest that *I. triloba* and *I. trifida* have the potential to improve sweetpotato resilience in dry environments and should be included in introgression breeding programs of this crop. Furthermore, *I. splendor-sylvae*, *I. ramosissima*, *I. tiliacea*, and wild *I. batatas* were the most productive species studied but given the genetic barriers to interspecific hybridization between these species and sweetpotato, we suggest that further genetic and metabolic studies be conducted on them. Finally, this study proposes a promising method for improving drought tolerance based on potential stress-memory induction, which is applicable both for wild species and crops.

## Introduction

In 2018, sweetpotato [*Ipomoea batatas* (L.) Lam.] was the root crop with the second highest global production after cassava [*Manihot esculenta* Crantz] (FAO, 2020). Sweetpotato is highly nutritious and outperforms most carbohydrate-based foods in terms of vitamins, minerals, dietary fiber, and total protein, making it a priority in crop-based strategies to enhance global food and nutrition security (Woolfe, 1992; Motsa et al., 2015). Sweetpotato is also known for its ability to grow in drought-prone soils with low external input (fertilizer and pesticide) requirements, while thanks to a short growing cycle, it is also recognized as an excellent crop for post-crisis (hurricane, flooding, refugee settlement, etc.) situations (Bradbury and Holloway, 1987; Mwanga and Ssemakula, 2011; Mekonnen et al., 2015). However, a range of biotic and abiotic stress factors such as viruses, weevils, and severe drought, are inhibiting farmers’ ability to achieve sweetpotato’s full yield potential (Valverde et al., 2007; Mwanga and Ssemakula, 2011; Agili and Nyende, 2012; Kivuva et al., 2015). Moreover, climate change’s negative effect on sweetpotato productivity is of rising concern in regions affected by increasing global temperatures, such as tropical and subtropical areas of Sub-Saharan Africa (Low et al., 2009; Schulze, 2011; Knox et al., 2012). Resilient sweetpotato varieties could play an extremely important food and nutrition security role in the developing world, one that will increase in importance as the availability of good cropping areas becomes more limited.

Series *Batatas* [*Ipomoea* series *Batatas* (Choisy) D. F. Austin] is a subdivision within *Ipomoea*, the largest genus in the morning glory (Convolvulaceae) family. This group includes the cultivated hexaploid sweetpotato [*I. batatas* (L.) Lam.], wild tetraploid *I. batatas* (L.) Lam. (Ozias-Akins and Jarret, 1994), and 15 closely related wild species (Austin, 1978; McDonald and Austin, 1990; Austin et al., 1993; Wood et al., 2015; Wood et al., 2020). The wild species include *Ipomoea trifida* (H.B.K.) G. Don, *Ipomoea cordatotriloba* Dennstedt, *Ipomoea cynanchifolia* Meisn., *Ipomoea grandifolia* (Dammer) O’Donell, *Ipomoea lacunosa* L., *Ipomoea leucantha* Jacquin, *Ipomoea littoralis* Blume, *Ipomoea ramosissima* (Poir.) Choisy, *Ipomoea splendor-sylvae* House, *Ipomoea tabascana* McDonald and Austin, *Ipomoea tenuissima* Choisy, *Ipomoea tiliacea* (Willd.) Choisy in D. C., *Ipomoea triloba* L., *Ipomoea lactifera* J.R.I. Wood and Scotland, and *Ipomoea australis* (O’Donell) J.R.I. Wood & P. Muñoz. Since sweetpotato crop wild relatives (SP-CWR) are well adapted to diverse, even extreme environmental conditions (Iwanaga, 1988; Guarino and Lobell, 2011), they are considered a prominent genetic resource to improve both biotic and abiotic stress tolerance in cultivated crops (Iwanaga, 1988; Komaki, 2004; Nimmakayala et al., 2011). However, Khoury et al. (2015) pointed out that the full potential offered by SP-CWR, especially in terms of drought tolerance, is far from being fully exploited.

From a physiological point of view, drought tolerance encompasses all mechanisms that enable plants to avoid or tolerate dehydration, such as maintenance of cell turgor and water uptake, and reduction of water loss, among others (Fischer and Maurer, 1978; Turner, 1986; Turner, 1997). In sweetpotato, several physiological traits have been used for screening drought tolerant genotypes such as chlorophyll concentration (Mbinda et al., 2018; Mbinda et al., 2019), canopy cover (Laurie et al., 2014), leaf temperature (Laurie et al., 2014; Rukundo et al., 2017; Low et al., 2020), and ^13^C discrimination (Low et al., 2020). The latter has been reported as one of the most accurate criteria for selecting drought tolerant genotypes (Tuberosa, 2012; Low et al., 2020) since it is a good indicator of stomatal conductance (Condon et al., 2004), water use efficiency (Turner, 1997), and photosynthetic performance (Jefferies and Mackerron, 1997; Dawson et al., 2002).

Previous studies in other plants such as *Arabidopsis thaliana* (Ling et al., 2018; Serrano et al., 2019), potato (Watkinson et al., 2006; Ramírez et al., 2015a), cassava (Cayón et al., 1997), wheat (Ahmed et al., 2016), and grasses (Walter et al., 2011) have shown that a previous exposure to stress in early development stages “prepares” the plant for a subsequent exposure to the same stress. This behavior, known as stress memory, is achieved through accumulation of transcription factor or signaling proteins and epigenetic changes, which are later translated into an enhanced physiological response (Bruce et al., 2007). In this study, we tested potential short-term memory occurrence, which involves tolerant responses within the plant life cycle to previous stress periods (Conrath et al., 2006; Bruce et al., 2007), as opposed to transgenerational memory, in which the tolerant response is inherited by the next plant generation (Han and Wagner, 2014). Wild species are considered to be possible sources of useful genes/alleles related to stress tolerance as they have evolved under natural selection to survive consecutives periods of climate extremes (Sharma et al., 2013). In the present study, we focused on the previously described physiological traits to determine potential stress memory induction in SP-CWR.

Farmers have traditionally selected drought tolerant genotypes by choosing those genotypes that maintained high yield under drought stress conditions. However, this selection strategy may penalize resilience or production stability, as shown by Blum (1996), who found that some genotypes with high tolerance presented low yield performance in the absence of stress conditions. Fernandez (1992) classified plants into four groups based on yield performance in stress and non-stress conditions: Group A (accessions expressing uniform superiority in both stress and non-stress conditions), Group B (accessions presenting good performance only under potential conditions and not under stress conditions), Group C (accessions expressing a relatively higher performance only under stress), and Group D (accessions with poor performance in both environments). Recently, Thiry et al. (2016) proposed a yield-based definition of drought tolerance in terms of resilience and productivity of crops useful to facilitate the classification of genotypes for breeding programs. These authors stated that the resilience capacity index (RCI) expresses the yield decrease of genotypes under stress within a population, compared with yield potential conditions, whereas the production capacity index (PCI) expresses the mean production of genotypes under both stressed and non-stressed conditions within a population. Moreover, a genotype could be classified in any of Fernandez’s (1992) Groups with those genotypes scoring the highest values being the most drought tolerant ones. Using combined indices such as resilience and productivity simultaneously in sweetpotato breeding programs would reduce costs and save time.

The aim of this study was i) to determine potential short-term memory induction in SP-CWR and its manifestation in ecophysiological traits like senescence, foliar area, leaf-minus-air temperature, and leaf ^13^C discrimination and ii) to identify the memory-induced physiological mechanisms associated with the development of drought tolerance (in terms of resilience and productivity) in SP-CWR in order to identify new sources for breeding towards improved drought tolerance in sweetpotato.

## Material and Methods

### Experimental Conditions and Management

A pot experiment was conducted under greenhouse conditions from July 13^th^ to December 18^th^, 2018 at the experimental station of the International Potato Center (CIP) in San Ramón, Junín, Peru (11° 7’ 39.3” S, 75°21’ 23.4” W, 850 m a.s.l.). The station is located in the mountainous Amazon area of central Peru. The region is characterized by a rainy, warm, and very humid climate (SENAMHI, 2020) with an average annual maximum temperature, average annual solar radiation and annual precipitation of 30.8 ± 0.46°C, 34.2 ± 1.51 MJ m^-2^ day^-1^, and 1,294 mm, respectively (data from 2019, CIP–San Ramón weather station). Pots were distributed in two neighboring screenhouses covered with an anti-aphid mesh with an opening size of 0.26 × 0.82 mm. The screenhouses were also covered with a translucid plastic and a black mesh (hole size of 2 × 2 mm) to prevent high radiation stress and provide shelter from rain. During the study period, the average relative humidity, and maximum and minimum temperatures were 81.2 ± 0.96%, 32.7 ± 0.47°C, and 19.8 ± 0.21°C respectively. The average solar radiation and maximum vapor pressure deficit were 18.5 ± 0.94 MJ m^-2^ day^-1^ and 2.7 ± 0.13 kPa (**Supplementary Table S1**). These variables were measured every 5 min. Temperature, relative humidity (S-THB-M008 model), and solar radiation (S-LIB-M003 model) sensors were recorded with a HOBO U30 datalogger (Onset Computer Corporation, Bourne, MA, USA).

On July 13^th^ eight seeds per accession were scarified individually and placed on moist filter paper in petri dishes. After germination seedlings were planted in peat pellets (Jiffy Products Ltd., Canada) for 15 days to promote root development. Subsequently, plantlets were transferred to pots (6.4 L) filled with 6.5 kg of a 2:1 mixture of sand and peat-based substrate (PRO-MIX, Premier Tech Horticulture, Canada). All pots were randomized regularly i.e. the positions of individual plants within the greenhouses were changed to avoid a significant effect of pot positions on the measured traits. In each screenhouse two HOBO U30 datalogger were placed at different positions (one near the central isle and another near the outside screen wall) to monitor temperature and humidity gradients.

Each pot received seven fertilizer applications scheduled every 2 weeks using 0.51 g N, 0.78 g P_2_O_5_, and 0.60 g, K_2_O (Peters Professional ICL Ltd., Israel). To control thrips and whitefly, folding traps with pheromones were installed and insecticides (Ocaren, active ingredient: profenofos and fipronil, Interoc S.A., Peru and Vertimec, active ingredient: abamectina; Farmex S.A., Peru) were applied at a dose rate of 1 mL L^-1^ when necessary. Since the wild species included in this study are twiners, a wire-made spiral welded to three vertical rods was placed in each pot to support the plants, in order to facilitate vertical growth and the expansion of foliar area.

### Plant Material

Fifty-nine accessions belonging to the *Ipomoea* series *Batatas* (Choisy) D. F. Austin, the closest wild relatives of sweetpotato, were selected from the SP-CWR collection of CIP’s genebank (**Table 1**). The plant material included four accessions of cultivated hexaploid *I. batatas* (L.) Lam. (hereinafter “sweetpotato”) namely “Beauregard,” “Tanzania,” and two accessions deriving from crosses between the two varieties: “B×T” (Wu et al., 2018). Also, two accessions of wild tetraploid (4x) *I. batatas* (L.) Lam. (Ozias-Akins and Jarret, 1994) and 53 accessions encompassing 10 species of the series *Batatas* were included. The wild relative species assessed in this study were: *I. australis* (O’Donell) J.R.I. Wood & P. Muñoz (5), *I. cordatotriloba* Dennstedt (1), *I. cynanchifolia* Meisn. (3), *I. grandifolia* (Dammer) O’Donell (5), *I. leucantha* Jacquin (2), *I. ramosissima* (Poir.) Choisy (8), *I. splendor-sylvae* House (3), *I. tiliacea* (Willd.) Choisy (2), *I. trifida* (H.B.K.) G. Don (16), and *I. triloba* L. (8) (Khoury et al., 2015; Wood et al., 2020). The basic chromosome number of all accessions in this study is x = 15. While most SP-CWRs of our taxon sample are diploid (2n = 2x = 30), four accessions are tetraploid (2n = 4x = 60) and the cultivated sweetpotato accessions are hexaploid (2n = 6x = 90) (G. Rossel, CIP, pers. comm.) (**Table 1**). The taxon sample of the wild species focused on Central and South American species and considered geographic distribution range and morphological variation between accessions of the same species.

**Table 1:** Sweetpotato crop wild relatives [*Ipomoea* series *Batatas* (Choisy) D. F. Austin] accessions from CIP’s genebank used in this study, with chromosome number and ploidy (basic chromosome number: x=15), country of origin and biological status for each of them.

Accession number	Species	Chromosome number and ploidy	DOI	Origin	Biological status	Groups
CIP 460360	*I. australis*	2n = 2x = 30^†^	10.18730/82SY	PRY	W	B
CIP 460296	*I. australis*	2n = 2x = 30^†^	10.18730/80T4	ARG	W	B
CIP 460345	*I. australis*	2n = 2x = 30^†^	10.18730/82AF	PRY	W	D
CIP 460585	*I. australis*	2n = 2x = 30^†^	10.18730/89GQ	ARG	W	D
CIP 460164	*I. australis*	2n = 2x = 30^†^	10.18730/7Y5Y	PRY	W	D
CIP 430434	*I. batatas*	2n = 4x = 60^†^	10.18730/852X	JAP	W	B
CIP 460577	*I. batatas*	2n = 4x = 60^†^	10.18730/898F	ECU	TC	B
CIP 440132	*I. batatas* “Beauregard”	2n = 6x = 90^*^	10.18730/65R1	USA	AC	B
CIP 440166	*I. batatas* “Tanzania”	2n = 6x = 90^*^	10.18730/66NY	UGA	TC	B
CIP 105269.232	*I. batatas* “B×T”	2n = 6x = 90^*^	10.18730/SK7J$	PER	Br	B
CIP 113641.086	*I. batatas* “B×T”	2n = 6x = 90^*^	10.18730/SK7K=	PER	Br	D
CIP 460077	*I. cordatotriloba*	2n = 2x = 30^†^	10.18730/7W75	MEX	W	B
CIP 460149	*I. cynanchifolia*	2n = 2x = 30^†^	10.18730/7XTK	BRA	W	B
CIP 460556	*I. cynanchifolia*	2n = 2x = 30^†^	10.18730/88M*	BRA	W	D
CIP 460555	*I. cynanchifolia*	2n = 2x = 30^†^	10.18730/88KZ	BRA	W	D
CIP 460610	*I. grandifolia*	2n = 2x = 30^†^	10.18730/89QY	BRA	W	B
CIP 460201	*I. grandifolia*	2n = 2x = 30^†^	10.18730/7YZK	ARG	W	D
CIP 460583	*I. grandifolia*	2n = 2x = 30^†^	10.18730/89EN	URU	W	D
CIP 460452	*I. grandifolia*	2n = 2x = 30^†^	10.18730/85MA	ARG	W	D
CIP 460337	*I. grandifolia*	2n = 2x = 30^†^	10.18730/8238	PRY	W	D
CIP 460619	*I. leucantha*	2n = 2x = 30^†^	10.18730/89T~	COL	W	B
CIP 460204	*I. leucantha*	2n = 2x = 30^†^	10.18730/7Z2P	ARG	W	D
CIP 460028	*I. ramosissima*	2n = 2x = 30^†^	10.18730/7V58	ECU	W	B
CIP 460032	*I. ramosissima*	2n = 2x = 30^†^	10.18730/7V9C	BOL	W	B
CIP 460566	*I. ramosissima*	2n = 2x = 30^†^	10.18730/88Y5	PER	W	B
CIP 460567	*I. ramosissima*	2n = 2x = 30^†^	10.18730/88Z6	PER	W	B
CIP 460005	*I. ramosissima*	2n = 2x = 30^†^	10.18730/7THS	PER	W	B
CIP 460047	*I. ramosissima*	2n = 2x = 30^†^	10.18730/7VNR	PER	W	B
CIP 460722	*I. ramosissima*	2n = 2x = 30^†^	10.18730/8B0$	ARG	W	B
CIP 460036	*I. ramosissima*	2n = 2x = 30^†^	10.18730/7VCF	BOL	W	D
CIP 460131	*I. splendor-sylvae*	2n = 2x = 30^†^	10.18730/7XE7	MEX	W	B
CIP 460373	*I. splendor-sylvae*	2n = 2x = 30^†^	10.18730/8355	NIC	W	B
CIP 460383	*I. splendor-sylvae*	2n = 2x = 30^†^	10.18730/83FF	NIC	W	B
CIP 460528	*I. tiliacea*	2n = 4x = 60^†^	10.18730/87V7	CUB	W	B
CIP 460531	*I. tiliacea*	2n = 4x = 60^†^	10.18730/87YA	CUB	W	B
CIP 460663	*I. trifida*	2n = 2x = 30^†^	10.18730/8A9B	MEX	W	C
CIP 113735.283	*I. trifida*	2n = 2x = 30^†^	10.18730/SK7P1	PER	Br	C
CIP 107665.9	*I. trifida*	2n = 2x = 30^*^	10.18730/SK7MU	PER	W	C
CIP 460026	*I. trifida*	2n = 2x = 30^†^	10.18730/7V36	COL	W	D
CIP 460430	*I. trifida*	2n = 2x = 30^†^	10.18730/84YS	CUB	W	D
CIP 460377	*I. trifida*	2n = 2x = 30^†^	10.18730/8399	NIC	W	D
CIP 460745	*I. trifida*	2n = 2x = 30^†^	10.18730/8BDA	GUA	W	D
CIP 460429	*I. trifida*	2n = 2x = 30^†^	10.18730/84XR	NIC	W	D
CIP 460096	*I. trifida*	2n = 2x = 30^†^	10.18730/7WKH	VEN	W	D
CIP 460195	*I. trifida*	2n = 2x = 30^†^	10.18730/7YSD	VEN	W	D
CIP 460022	*I. trifida*	2n = 2x = 30^†^	10.18730/7V03	COL	W	D
CIP 113735.258	*I. trifida*	2n = 2x = 30^*^	10.18730/SK7N0	PER	Br	D
CIP 113735.302	*I. trifida*	2n = 2x = 30^*^	10.18730/SK7Q2	PER	Br	D
CIP 113735.329	*I. trifida*	2n = 2x = 30^*^	10.18730/SK7R3	PER	Br	D
CIP 107665.19	*I. trifida*	2n = 2x = 30^*^	10.18730/SK7H~	PER	W	D
CIP 460021	*I. trifida*	2n = 2x = 30^†^	10.18730/7TZ2	VEN	W	D
CIP 460309	*I. triloba*	2n = 2x = 30^†^	10.18730/817H	PRY	W	B
CIP 460116	*I. triloba*	2n = 2x = 30^†^	10.18730/7X5=	COL	W	C
CIP 460560	*I. triloba*	2n = 2x = 30^†^	10.18730/88RU	PER	W	D
CIP 460052	*I. triloba*	2n = 2x = 30^†^	10.18730/7VSW	VEN	W	D
CIP 460093	*I. triloba*	2n = 2x = 30^†^	10.18730/7WJG	DOM	W	D
CIP 460517	*I. triloba*	2n = 2x = 30^†^	10.18730/87H$	ECU	W	D
CIP 460784	*I. triloba*	2n = 2x = 30^†^	10.18730/8C1Y	JAM	W	D
CIP 460078	*I. triloba*	2n = 2x = 30^†^	10.18730/7W86	MEX	W	D

Groups were defined based on clustering analysis (see **Figure 4**). Groups B, C and D represent clusters I, II and III, respectively.

W, wild; TC, traditional cultivar/landrace; AC, advanced or improved cultivar; Br, breeding line. ^†^(G. Rossel, CIP, pers. comm.). ^*^ Wu et al. (2018).

### Drought Priming Process

**Figure 1:**
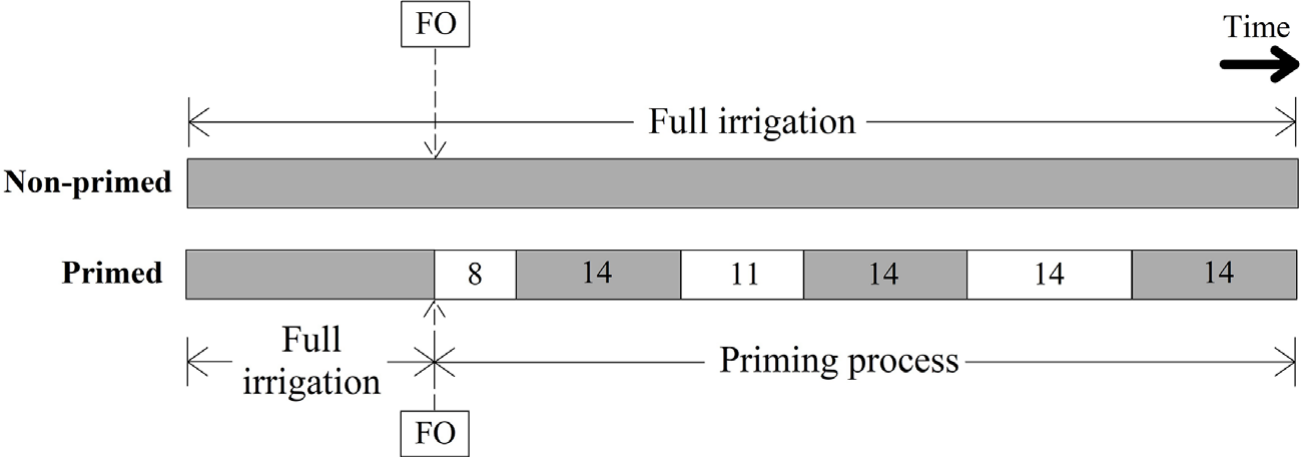
Timeline representation for the watering treatments per accession: non-primed plants (no water restriction) and primed plants (water restriction periods). Gray and white blocks mean substrate watering with full irrigation or no irrigation, respectively. Duration of every period during the priming process is indicated in days within the blocks. The priming process started after flowering onset (FO).

Eight individual plants per accession were distributed at random in both greenhouses. Four individual plants per accession (i.e. four replications) were randomly assigned to the two experimental treatments. Previous stress memory studies by Liu and Charng (2012); Ling et al. (2018) and Serrano et al. (2019) were used to guide the experimental design of this study. Treatments consisted of a series of water restriction periods of increasing length (priming), *versus* full irrigation (non-priming). After transplantation of seedlings into pots, all pots were watered until field capacity (0.32 w/w) three times per week, following the protocol of Rolando et al. (2015), until harvest. Non-primed plants received full irrigation during the entire experimental cycle while plants undergoing the water restriction treatment were watered until flowering onset (FO) (**Figure 1**). Priming was initiated when 50% of samples conforming an accession started to exhibit flower buds (defined as FO occurrence) (Thiry et al., 2016). Two different starting dates were established for early-flowering (20 accessions) and late-flowering (18 accessions) or non-flowering (21 accessions) accessions (**Supplementary Table S2**). Three priming events, or water restriction periods, were carried out with 8, 11, and 14 days of total water restriction. Each of these water restricted periods was followed by a recovery period of 14 days in which plants were irrigated until field capacity (**Figure 1**). Therefore, the priming process consisted of three water restriction periods: the first one started at FO whereas the second and third priming events were initiated at 68 and 94 days after transplanting (DAT) for early as well as 94 and 122 DAT for late and non-flowering accessions, respectively.

Drought stress responses were measured at the end of the first water restriction period to corroborate if 8 days was a sufficiently long period to induce a significant drought stress. For this purpose, maximum stomatal conductance at saturated light (g_s_max_) was measured (*sensu* Medrano et al., 2002) in one young and sun-exposed leaf of eight plants of 19 randomly selected accessions using a portable photosynthesis system (LI-6400TX, LI-COR, Nebraska, USA). Measurements were carried out during early morning hours from 6:00 to 10:00 h local time. The following micro-environmental parameters were recorded: photosynthetic active radiation = 1,200 µmol m^-2^ s^-1^, CO_2_ concentration = 400 ppm, and atmospheric humidity = 50%.

### Ecophysiological Measurements to Determine Potential Short-Term Memory Occurrence

Chlorophyll concentration (Chl_SPAD_) of all accessions was measured using a portable chlorophyll meter (SPAD-502 Plus, Konica Minolta Inc., Osaka, Japan) on 17 occasions during the study period. An average of four readings from four young and sun-exposed leaves were taken per plant. Senescence (S) was estimated on each plant as the slope generated by fitting Chl_SPAD_ (from maximum leaf greenness to harvest) *vs.* time on a linear function (Li et al., 2019). Assuming that an extended senescence delay (high S) is associated with a higher probability of fixing more carbon during the lifespan, short-term memory proxy’s (STM) effect on S (STM_S_) was calculated as follows:

(1) $$STM_{S}=S_{pr}-S_{npr}$$

Where S_pr_ and S_npr_ are S average values for primed and non-primed plants, respectively. Regular assessments of foliar area (FA) were carried out on both primed and non-primed plants before and after each water restriction and recovery period by taking visible images (using a Nikon model D7000 camera, Nikon Corp., Japan) of each plant. Images were acquired following CIP procedures (PSE-CIP, 2013) and processed with Image Canopy software (Barreda et al., 2017) that allows the calculation of foliar area through an image segmentation technique separating healthy green vegetation from other components within a picture. To test the effect of STM on FA (STM_FA_) the following equation was calculated:

(2) $$STM_{FA}=\frac{FA_{\text{max}\_pr}}{FA_{\text{max}\_npr}}$$

Where FA_max_pr_ and FA_max_npr_ are FA average maximum value of the temporal assessments corresponding to primed and non-primed plants, respectively. Leaf temperature was measured radiometrically from 14:00 to 15:00 h following the protocols of Idso et al. (1981) and Rinza et al. (2019). Measurements were taken using an infrared thermometer (DT-882 model, CEM, China) during 14 measurement events throughout the study period. Air temperature was registered with four data loggers (HOBO U23 Pro v2 Temperature/Relative Humidity, Onset Computer Corporation, Bourne, MA, USA), two of which were located inside each greenhouse at canopy level. The difference between leaf and air temperature (dT) was calculated using average leaf temperature and average air temperature registered at the same time when leaf temperature was measured. STM effect on dT (STM_dT_) was calculated as follows:

(3) $$STM_{dT}=\frac{dT_{\text{min}\_pr}}{dT_{\text{min}\_npr}}$$

Where dT_min_pr_ and dT _min_npr_ are the average minimum dT over time reached by primed and non-primed plants of an accession. Finally, two composed leave samples per accession and treatment were collected at the end of each recovery period. Six assessments in total were carried out from FO until harvest at 66, 93, and 121 DAT for early flowering accessions and at 94, 121, and 148 DAT for late- or non-flowering accessions. Each composed sample consisted of 20 leaves—five young leaves per plant—which were oven dried at 60°C for 48 h (BLUE M Model POM-166EY, BLUE M Electronic Company, IL, USA). Dried leaves were milled with a ball miller (MBIX-100 model, MRC, Israel) and packed in tin capsules (Ramírez et al., 2015b). Capsules were sent to the Stable Isotope Facility at the University of Davis, USA, for carbon isotope composition (δ^13^C) analysis using a PDZ Europa ANCA-GSL elemental analyzer coupled to PDZ Europa 20-20 isotope ratio mass spectrometer (Sercon Ltd., Cheshire, UK). Leaf ^13^C discrimination (Δ) was calculated as described by Farquhar et al. (1989):

(4) $$\Delta \left(‰\right)=\left(\frac{\delta_{a}-\delta_{p}}{1+\delta_{p}}\right)\times 1000$$

Where δ_p_ is δ^13^C of the sample and δ_a_ is the δ^13^C of the atmospheric CO_2_, -8‰. STM effect on Δ (STM_Δ_) was estimated as follows:

(5) $$STM_{\Delta }=\frac{\Delta_{\text{max}\_pr}}{\Delta_{\text{max}\_npr}}$$

Where Δ_max_pr_ and Δ_max_npr_ are the maximum Δ over time for primed and non-primed plants within an accession, respectively. The STM occurrence was defined when primed plants’ response exceeded that of non-primed plants, i.e. when the STM value was higher than zero (STM>0) in the case of S in Equation 1, and higher than one (STM>1) in the case of FA, dT and Δ in Equation 2, 3, and 5, respectively. For all accessions, the priming process started at the same physiological stage (following recommendations from Thiry et al., 2016) so that all the ecophysiological indicators (S, A_max_, dT_min_, and Δ_max_) used to determine potential STM occurrence were comparable between accessions regardless of the starting date of the priming process.

### Drought Tolerance Indices and Statistical Analysis

At the end of the priming process, total biomass was harvested and subsequently oven dried at 60°C for 48 h to calculate the dry weight of total biomass of primed (Y_pr_) and non-primed (Y_npr_) plants. In this study, total biomass comprises above and below ground biomass and is generally expressed on a dry matter (DM) basis. The stress susceptibility index (SSI) (Fischer and Maurer, 1978) and the geometric mean productivity index (GMP) (Fernandez, 1992) were calculated as follows:

(6) $$SSI=\frac{1-(Y_{pr}/Y_{npr})}{1-(\bar{Y_{pr}}/\bar{Y_{npr}})}$$

(7) $$GMP=\sqrt{Y_{pr}\times Y_{npr}}$$

Where Y_pr_ and Y_npr_ are the average total biomass (DM) production of the primed and non-primed plants, respectively, and $\bar{Y_{pr}}$ and $\bar{Y_{npr}}$ are the overall average total biomass (DM) production of the primed and non-primed plants, respectively. SSI and GMP of a genotype were estimated as the resilience capacity index (RCI) and the production capacity index (PCI) following the score values method described by Thiry et al. (2016) (**Supplementary Appendix A**). For of both score indices (RCI and PCI), a score value of 1 means high susceptibility whereas a score of 10 represents high drought tolerance.

A repeated measures analysis of variance (rmANOVA) was run to evaluate the effects of the treatments (between subjects factor) and time (within subjects factor) on assessed ecophysiological variables (SPAD, FA and dT, except for Δ, due to its composed samples). A one-way ANOVA was used to assess differences (at p < 0.05) among accessions in total dry weight (Y). A t-student test was used to identify significant differences between treatments for every physiological evaluation of SPAD, FA, dT, Δ, and Y. A Pearson correlation analysis was performed to evaluate the relationship between STM traits and both RCI and PCI. A principal component analysis (PCA) was run to analyze the accessions ordination through the association of STM trait effects and RCI, as well as PCI. Finally, a cluster analysis following the Ward’s method was computed using the R package “FactoMineR” (Lê et al., 2008) to classify all accessions into any of the four Groups of plants described by Fernandez (1992). All above described tests were performed with RStudio software (R Core Team, 2019).

## Results

### Short-Term Memory Occurrence

**Figure 2:**
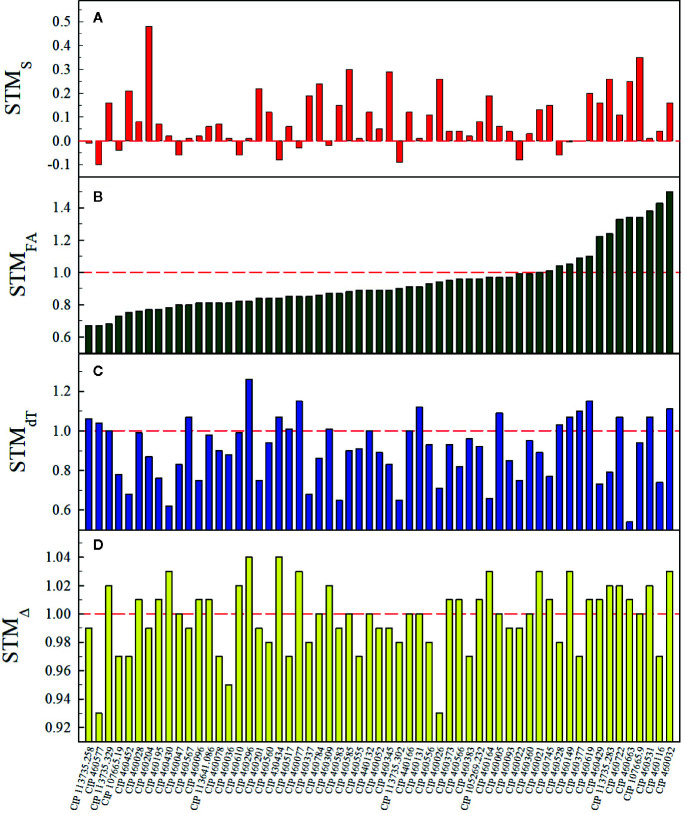
Short-term memory (STM) values per accession for senescence (STM_S_) **(A)**, foliar area (STM_FA_) **(B)**, leaf-minus-air temperature (STM_dT_) **(C)** and leaf ^13^C discrimination (STM_Δ_) **(D)**. Red dashed line indicates STM occurrence.

**Figure 3:**
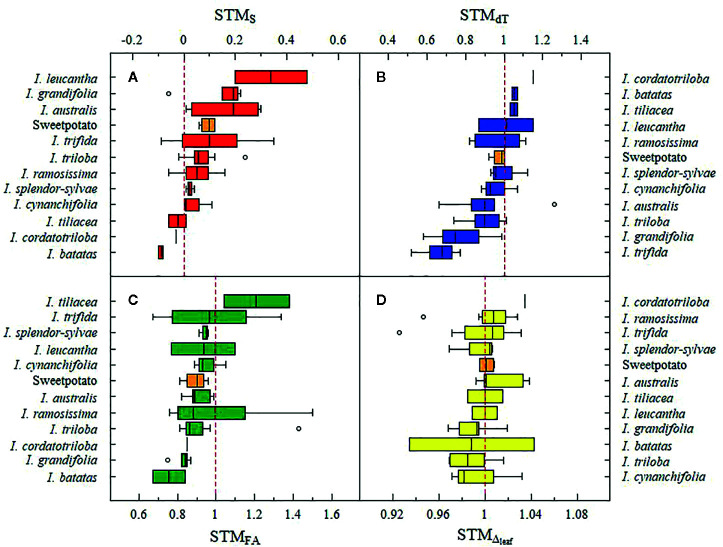
Boxplot of short-term memory (STM) values per crop wild relatives (CWR) species and sweetpotato cultivars (orange boxplots) for senescence (STM_S_) **(A)**, foliar area (STM_FA_) **(B)**, leaf-minus-air temperature (STM_dT_) **(C)** and leaf ^13^C discrimination (STM_Δ_) **(D)**. Red dashed line indicates the STM occurrence. In each specie’s boxplot, black line represents the median value. Boxplot contains the variation between 25 and 75% and gray circles are the outlier values.

Chl_SPAD_, FA, dT, and Δ values ranged between 10.3–51.4 SPAD units, 0.0–1,537.3 cm^2^, −12.2 – 4.1°C, and 20.4–25.3‰, respectively. Effects of the water treatment for Chl_SPAD_, FA, and dT were significant (p < 0.05) for 33.9, 81.4, and 81.4% of the total accessions and the effect of time was significant in all accessions for Chl_SPAD_, FA, and dT. The percentage of accessions with a significant (p < 0.05) interaction between watering treatments and time for Chl_SPAD_, FA, and dT were 93.2, 98.3, and 78.0%, respectively (**Supplementary Table S3**). At the end of the first water restriction period, at 53 DAT, primed plants had 78.6% less average g_s_max_ than non-primed plants (0.02 ± 0.03 and 0.10 ± 0.04 mol H_2_O m^-2^ s^-1^ for primed and non-primed plants, respectively) of the 19 assessed accessions. Moreover, 16 accessions (81.3%) presented primed plants with average g_s_max_ below 0.05 mol H_2_O m^-2^ s^-1^ (**Supplementary Table S4**). STM occurrence was detected in 23.7, 28.8, 50.8, and 81.4% of the total number of accessions for FA, dT, Δ, and S, respectively (**Figure 2**). For STM_S_, *I. leucantha* presented the highest value (0.48 for CIP 460204) and *I. trifida* had the widest range (−0.09 – 0.35) (**Figure 3A**) while for STM_FA_, *I. ramosissima* presented the highest value (1.5 for CIP 460032) as well as the widest range (0.76–1.5) (**Figure 3B**). *I. australis* showed both the highest STM_dT_ value (1.26 for CIP 460296) and the widest STM_dT_ range (0.66–1.26) (**Figure 3C**) whereas *I. batatas* had the highest STM_Δ_ value (1.04 for CIP 430434) and *I. trifida* the widest STM_Δ_ range (0.93–1.03) (**Figure 3D**). The raw data and RGB images of each plant are available online (Guerrero-Zurita et al., 2020).

### Drought Tolerance, Resilience, and Productivity Under Drought and Their Relationship With Short-Term Memory Occurrence

SSI and GMP values ranged between −0.83 – 1.74 and 7.56–78.1, respectively (**Supplementary Table S5**). High coefficients of determination between the original values for SSI and GMP and their RCI and PCI score indices were obtained (R^2^ = 0.970 for SSI *vs.* RCI and R^2^ = 0.974 for GMP *vs.* PCI) indicating that score indices can be used as a surrogate of their original index value. The highest RCI were found in CIP 460116 (8.0), CIP 107665.9 (7.5), and CIP 460663 (7.0) while *I. triloba* had the highest range of values (3.5–8.0) followed by *I. trifida* (2.0–7.5) and *I. grandifolia* (1.75–5.25). Regarding PCI, the highest values were found in CIP 440166 (9.75), CIP 460131 (6.0), and CIP 440132 (5.75) while sweetpotato cultivars presented the highest range of scores (2.75–9.75) followed by *I. splendor-sylvae* (4.25–6) and *I. ramosissima* (2.5–5.75). Moreover, *I. cynanchifolia*, *I. australis*, *I. grandifolia*, *I. trifida*, and *I. triloba* had higher RCI than PCI, whereas *I. leucantha*, *I. ramosissima*, *I. cordatotriloba*, *I. tiliacea*, wild *I. batatas*, *I. splendor-sylvae*, and the sweetpotato cultivars had higher PCI than RCI.

STM_dT_ showed a significant positive and negative correlation with PCI (r = 0.52) and RCI (r = −0.30), respectively, whereas STM_FA_ was the trait with the highest positive correlation with RCI (r = 0.38) (**Table 2**). The first three components of the PCA represented 72.7% of the total variance (**Table 3**). The first component was mainly explained by STM_S_ and RCI with a negative effect and STM_dT_ and PCI with a positive effect. STM_FA_ showed a higher weight in the second principal component. The third principal component was mainly explained by STM_dT_ with positive effect (**Table 3**).

**Table 2:** Pearson correlation coefficients matrix among short-term memory (STM) traits and drought tolerant indices.

	STM_S_	STM_FA_	STM_dT_	STM_dT_	RCI
STM_FA_	0.22			
STM_dT_	-0.26^∗^	-0.01			
STM_Δ_	0.01	0.20	0.20		
RCI	0.21	0.38^∗∗^	-0.30^∗^	-0.14	
PCI	-0.25	0.13	0.52^∗∗^	0.19	-0.27^∗^

RCI, resilience capacity index; PCI, production capacity index. See STM traits abbreviations in **Figure 2**. ^∗∗^p < 0.01, ^∗^p < 0.05.

**Table 3:** Extracted components from Principal Component Analysis based on the ordination of short-term memory (STM) traits, drought tolerance resilience (RCI), and productivity (PCI).

Variable	PC 1	PC 2	PC 3
STM_S_	-0.55	0.30	0.46
STM_FA_	-0.21	0.85	-0.22
STM_dT_	0.77	0.21	-0.14
STM_Δ_	0.33	0.54	0.61
RCI	-0.66	0.37	-0.45
PCI	0.73	0.34	-0.25
Eigen-value	2.03	1.42	0.91
TCV (%)	31.86	57.47	72.72

TCV, total cumulative variance; PC, principal component. See STM traits abbreviations in **Figure 2**.

**Figure 4:**
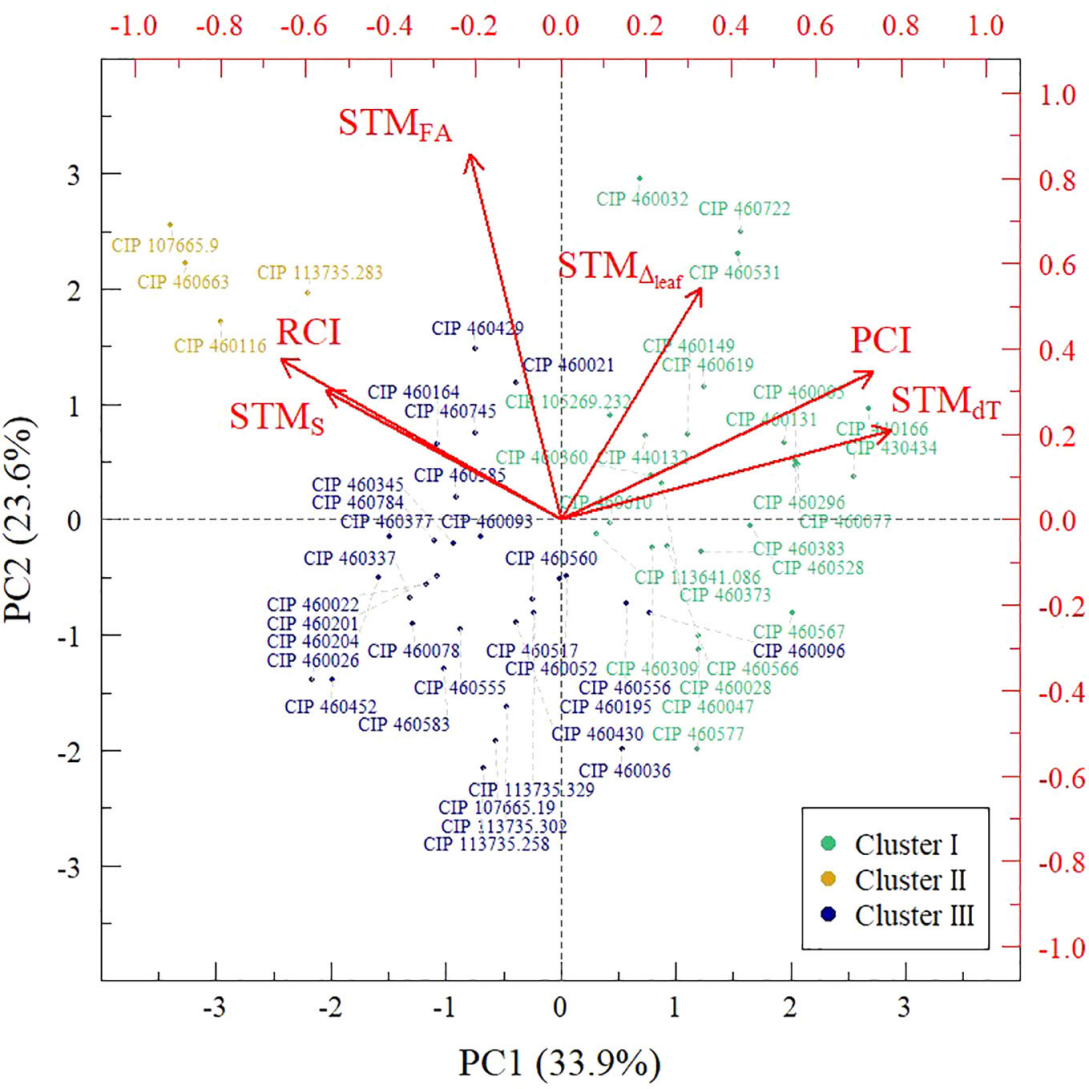
Clustering analysis and 2D ordination of sweetpotato cultivars and its CWR species based on Principal Component Analysis for component loadings (STM traits, PCI and RCI). Clusters I, II and III correspond to Fernandez's (1992) Groups B, C, and D, respectively.

**Figure 5:**
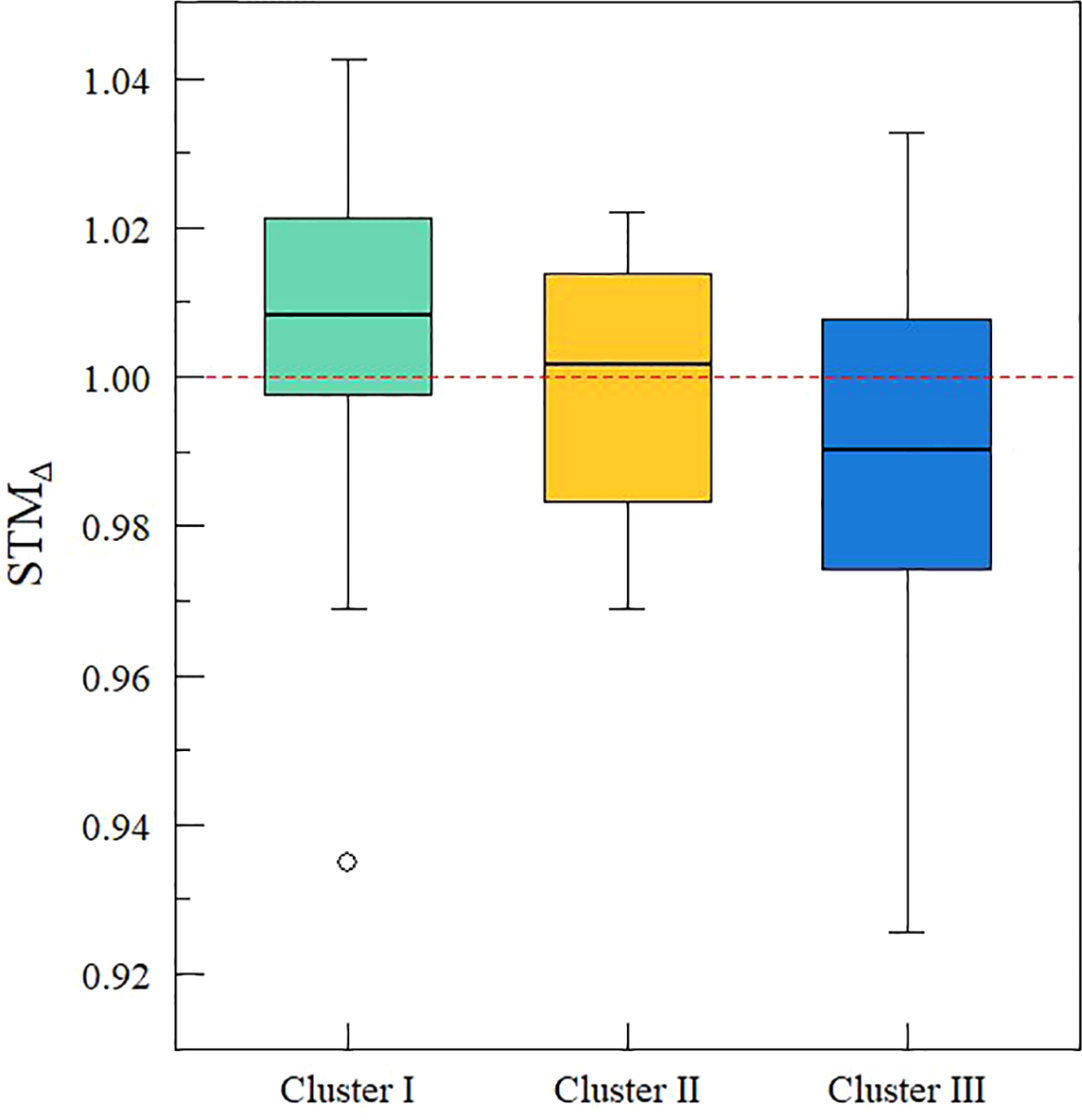
Boxplot of short-term memory (STM) effect on leaf ^13^C discrimination (STM_∆_) for each cluster. Red dashed line indicates the STM occurrence (STM_Δ_ > 1). In each cluster’s boxplot, black line represents the median value. Boxplot contains the variation between 25 and 75% and gray circles are the outlier values.

Clustering analysis grouped the accessions into three clusters: I, II, and III (**Figure 4**). Cluster I, II, and III contained 24, 4, and 31 accessions, respectively (**Table 1**). When analyzing the average response per variable obtained in each group, cluster I had the highest average for STM_dT_, STM_∆_, PCI, and Y_npr_ (1.03 ± 0.1, 1.01 ± 0.0, 4.39 ± 1.7, and 49.1 ± 16.4 g, respectively) whereas cluster II was highest for STM_S_, STM_FA_, and RCI (0.23 ± 0.1, 1.34 ± 0.1, and 7.0 ± 1.1, respectively) (**Table 4**). Accessions belonging to cluster III displayed the lowest average response for all traits (**Table 4**). Cluster I contained the highest percentage of accessions with STM_∆_ occurrence (70.8%) followed by cluster II (50%) and cluster III (35.5%) (**Figure 5**).

**Table 4:** Average value ± standard error of STM traits, drought tolerance indices and total biomass production for each cluster (I, II and III) from Principal Component Analysis.

	Cluster		
Variable	I	II	III
STM_S_	0.03 ± 0.01	0.23 ± 0.1	0.11 ± 0.1
STM_FA_	0.96 ± 0.2	1.34 ± 0.1	0.87 ± 0.1
STM_dT_	1.03 ± 0.1	0.63 ± 0.1	0.78 ± 0.1
STM_Δ_	1.01 ± 0.0	1.00 ± 0.0	0.99 ± 0.0
RCI	3.17 ± 1.0	7.0 ± 1.1	3.77 ± 1.0
PCI	4.39 ± 1.7	1.81 ± 0.6	2.04 ± 0.9
Y_npr_ (g)	49.1 ± 16.4	17.6 ± 4.8	24.5 ± 9.3
Y_pr_ (g)	25.9 ± 9.4	16.6 ± 2.6	14.4 ± 5.0

Y_npr_, total biomass production of non-primed treatment; Y_pr_, total biomass production of primed treatment. See STM traits, RCI, and PCI abbreviations in **Figure 2**.

## Discussion

### Priming Induction Was Expressed Mainly in Senescence Delay and Photosynthetic Performance Traits

The highest Chl_SPAD_ values (51.4, 50.5, and 49.4 SPAD units for accessions CIP 440166, CIP 430434, and CIP 460528, respectively) observed in this study were higher than previously recorded in root and tuber crops such as sweetpotato (~42.0 SPAD units; Mbinda et al., 2018; Mbinda et al., 2019), cassava (47 SPAD units; Ogaddee and Girdthai, 2019), and potato (49 SPAD units; Rolando et al., 2015 and ~45 SPAD units; Ramírez et al., 2014). Also, the lower dT_min_ average value obtained here (−12.2 ± 0.7°C, for primed CIP 460531), was much lower than minimum dT values reported in potato (about −6°C; Stark et al., 1991) or alfalfa (approximately −10°C; Idso et al., 1981) under no-stress conditions. Given that the minimum value was achieved by primed plants in the recovery period of the third water restriction period, these results suggest an optimization in stomatal behavior as a result of the priming process. The highest Δ_max_ average value (25.3‰ for primed CIP 460583) obtained in this study in the third water restriction period, was higher than previously reported for sweetpotato (21.7 ‰, Zhang et al., 2015 and 23.6‰, Ramírez et al., 2017) or potato (23‰, Ramírez et al., 2015b).

Studies of stress memory response in *A. thaliana* (Ling et al., 2018; Serrano et al., 2019) found an improvement in heat-stress tolerance after a second priming event. The ecophysiological indicators (S, FA_max_, dT_min_, and Δ_max_) assessed in our study indicated an improvement in primed plants after the first water restriction period. Delayed senescence was the most important trait increasing after water restriction events in 81.4% of the genotypes (**Figure 2A**). Delayed senescence is the result of a stress-response mechanism characterized by slower chlorophyll degradation over time, in comparison to unstressed genotypes (Thomas and Howarth, 2000; Rivero et al., 2007; Abdelrahman et al., 2017). In both potato (Rolando et al., 2015) and sweet potato (Smit, 1997; Bararyenya et al., 2020), delayed senescence has been attributed to outstanding yield, since it extends the period of time in which the plant can fix carbon. On the other hand, primed plants from 23.7% of the total number of accessions (**Figure 2B**) increased their foliar area (i.e. higher aerial biomass than non-primed plants) after the first water restriction period. These findings are corroborated by previous studies in which higher green leaf area was observed during recovery from early season drought in tolerant genotypes (Rivero et al., 2007; Puangbut et al., 2009; Lewthwaite and Triggs, 2012). Foliar area—a trait prioritized in breeding programs (Lenis et al., 2006; De Souza et al., 2017)—has been correlated to light interception and use efficiencies (Legg et al., 1979; Zhu et al., 2008; De Souza et al., 2017).

Another important trait to be considered in breeding programs that seek to improve drought tolerance is canopy temperature (Obidiegwu et al., 2015). The ability of plants to cool their leaves *via* stomata while at the same time saving water has been correlated with drought tolerance (Blum and Arkin, 1984; Blum, 2005; Hirayama et al., 2006; Ramírez et al., 2015b). The leaf-minus-air temperature (dT) has thus been used as an index of plant water status (Takai et al., 2010; Tuberosa, 2012; Iseki et al., 2018) or as an indirect method of indicating stomatal conductance (Jackson et al., 1977; Hatfield, 1983; Rinza et al., 2019). Previous studies have demonstrated that genotypes with the lowest dT values (here dT_min_) had the highest transpiration and photosynthetic rates (Hirayama et al., 2006; Takai et al., 2010; Rukundo et al., 2017). These results are corroborated by our findings, in which primed plants from 17 accessions (**Figure 2C**) presented lower dT values in the second or third recovery period than those obtained by their respective non-primed plants throughout their lifespan. Our results suggest that a potential short-term memory induced cooling mechanism in primed plants enabled them to decrease leaf temperatures in response to hot and dry environments.

On the other hand, ^13^C discrimination integrates the photosynthetic performance throughout the period of leaf tissue synthetization (Jefferies and Mackerron, 1997; Dawson et al., 2002). According to literature, this trait is expected to decrease in drought conditions, due to a reduced discrimination against ^13^C by RuBisCO (Farquhar et al., 1989), and to partial recovery after re-watering (Xu et al., 2010; Ramírez et al., 2016; Silva-Díaz et al., 2020). In that respect, leaf ^13^C discrimination from primed plants, measured in every recovery period, reflects the effects of previous water restriction periods. Memory effects were determined by calculating the maximum leaf ^13^C discrimination (Δ_max_) reached by primed plants and comparing it with the one achieved by non-primed plants. Primed plants from 30 accessions (**Figure 2D**) mitigated the negative effects of the first water restriction period, and even reached a higher Δ_max_, at the end of the priming process, than non-primed plants grown under fully irrigated conditions suggesting a potential short-term memory occurrence. This improvement in photosynthetic performance has also been documented in potato (Ramírez et al., 2016; Silva-Díaz et al., 2020), wheat (Monneveux et al., 2005), and rice (Impa et al., 2005), but not on a level as high as observed in our study, induced by short-term memory in primed plants.

Of the four traits evaluated, the performance of senescence and photosynthesis were the most favorable following the priming process, which revealed their potential flexibility under stress memory induction. These results suggest that potential stress memory induction helps modify metabolic pathways related to chlorophyll degradation (Rivero et al., 2007; Abdelrahman et al., 2017) and photosynthesis recovery (Ramírez et al., 2016). Also, despite their corroborated relation to drought tolerance, FA and dT presented a lower percentage of STM occurrence, suggesting a more complex response enhanced by stress memory. Species such as *I. leucantha*, *I. australis* and *I. grandifolia* presented even higher STM_S_ than sweetpotato cultivars (**Figure 3A**), leading us to recommend the consideration of these CWR species for use in breeding programs. Regarding photosynthetic performance, all SP-CWR species contained at least one accession in which STM occurrence (**Figure 3D**) suggests the potential of this group of species for easy recovery in environments with prolonged drought seasons.

### A New Model for Drought Tolerance Improvement in Sweetpotato Based on Stress-Memory Induction and Wild Relatives

Resilience of a crop is the extent to which it is capable of surviving stress or other perturbations through physiological adaptations, with a minimum effect on yield (Holling, 1973; Trenbath, 1999). There is a differentiated capability of sweetpotato genotypes to allocate more carbon to above ground biomass than harvestable roots (e.g. Ejumula variety) under non-stress growth conditions (Coleman et al., 2006; Ramírez et al., 2017), however, the implications of this shifted resource allocation have not been explored yet. In our study, the capability to increase foliar area appears to be the main trait responsible for enhanced resilience (**Table 2**), an observation that is in agreement with previous studies on sorghum in which genotypes submitted to severe water restriction presented higher yields due to both increased leaf area and delayed senescence (Borrell et al., 2000). Moreover, in cassava, De Souza et al. (2017) highlight the importance of breeding for genotypes with increased leaf area to improve biomass production and drought response. Our results suggest that there is an association between delayed senescence and increased foliar area that elevated RCI response (**Figure 4**). In cereals, early-heading, which translates as a delayed senescence, is considered an escape strategy from adverse environmental conditions (Turner, 1979; Levitt, 1980; Dolferus, 2014) rather than representing a resilience response (Thiry et al., 2016). This study’s results suggest that delayed senescence is the cause of increased leaf area. This resilience response includes more and longer-living foliage, which provides more time and space to fix additional CO_2_ (Legg et al., 1979; Zhu et al., 2008; De Souza et al., 2017), and results in higher aerial biomass (Puangbut et al., 2009). However, the link between senescence delay and leaf biomass production is a relationship that should be explored more profoundly in the future.

Crop productivity has been associated with canopy temperature (e.g. dT) and the latter has been frequently used as a selection method for drought tolerance (Jackson et al., 1981; Jones, 2006; Takai et al., 2010; Zia et al., 2013). Some studies have shown that lower dT values are a consequence of higher stomatal conductance (Jackson et al., 1977; Hatfield, 1983; Rinza et al., 2019) and effective water uptake (Blum, 2009) representing the optimization of leaf thermoregulation and gas exchange. In our study, production capacity (PCI) was mainly related to STM_dT_ (**Table 2**) confirming the aforementioned results of other authors. The combination of dT and leaf ^13^C discrimination were associated with elevated PCI values (**Figure 4**) suggesting that optimized leaf thermoregulation and gas exchange is a consequence of improved photosynthetic performance and recovery (Jefferies and Mackerron, 1997; Dawson et al., 2002), which ultimately leads to higher levels of productivity in SP-CWR.

Three groups of accessions performed differently in this study. Accessions from cluster I, presented higher biomass production in non-stress (Y_npr_) than in water deficit conditions (Y_pr_) (**Table 4**). Accessions in this cluster correspond to Group B of Fernandez’s (1992) classification of plants based on biomass production (**Table 1**). Group B corresponds to Thiry et al. (2016) classification based on PCI and RCI, since accessions falling into group B also showed the highest average PCI value (4.39 ± 1.7). In cluster II, primed plants equaled the biomass production obtained from non-primed plants of the same accession (**Table 4**). This together with the highest average RCI value (7.0 ± 1.1), suggests that these accessions belong to Group C in Fernandez (1992) classification (**Table 1**). Cluster III of this study corresponds to Group D in Fernandez (1992) classification (**Table 1**) because the respective accessions had the lowest biomass production performance in both non-stress and stress conditions and the lowest PCI and RCI values (**Table 4**). Our results suggest that sweetpotato crop wild relatives possess outstanding physiological mechanisms to respond to both non-stress and water restriction scenarios. Primed SP-CWR species from cluster II (**Table 1**) such as *I. triloba* (CIP 460116) and *I. trifida* (CIP 107665.9, CIP 460663, and 113735.283) showed better biomass production performance under drought stress and hence could be considered for use in breeding programs (Thiry et al., 2016) to improve sweetpotato resilience, a trait much appreciated by breeders (Andrade et al., 2016). The aforementioned species coincide with previous studies (Iwanaga, 1988; Komaki, 2004; Zhang and Liu, 2005; Nimmakayala et al., 2011), which were based on biomass production under stress conditions but did not assess physiological performance. On the other hand, accessions from species such as *I. splendor-sylvae*, *I. ramosissima*, *I. tiliacea*, and wild *I. batatas* from cluster I could only be used as a potential genetic source of traits related to optimized leaf transpiration and photosynthetic performance.

The enhancement of drought tolerance in our study is also supported from a physiological point of view. Ramírez et al. (2015a) defined drought tolerance enhancement based on ^13^C discrimination, when primed plants presented higher ^13^C discrimination than non-primed plants. This physiological criterium matches our RCI-PCI drought tolerant genotypes from cluster I and II, as both of them contained the highest number of accessions with STM_∆_ occurrence (**Figure 5**). Therefore, we have demonstrated, experimentally, the existence of drought tolerance and, physiologically, the drought tolerance enhancement induced by short-term memory.

Due to ploidy differences of the taxa and other barriers to hybridization, interspecific hybridization between sweetpotato and related wild species is challenging and usually requires the employment of a pre-breeding approach. However, several authors have shown that the creation of interspecific hybrids is feasible using ovule culture (Kobayashi et al., 1994), somatic cell hybridization (Liu et al., 1994; Zhang et al., 2002; Yang et al., 2009), application of phytohormones in combination with controlled pollination (Cao et al., 2009), creation of synthetic hexaploids and triploids (Nishiyama et al., 1975; Shiotani and Kawase, 1987; Freyre et al., 1991), and interploidy hybridization (Orjeda et al., 1991). Successful production of hybrids between hexaploid sweetpotato and CWR species of the Batatas complex was reported for *I. trifida* (Orjeda et al., 1991; Kobayashi et al., 1994)*, I. triloba* (Kobayashi et al., 1994; Liu et al., 1994; Yang et al., 2009)*, I. lacunosa* (Kobayashi et al., 1994; Zhang et al., 2002)*, I. grandifolia* (Cao et al., 2009), *I. littoralis* (Nishiyama et al., 1975), and *I. leucantha* (Nishiyama et al., 1975). Moreover, modern breeding techniques (e.g. CRISPR/Cas9-mediated genome editing) offer promising options for introgression of genes from wild relatives into the hexaploid cultivated genepool which need to be further explored (Wang et al., 2019).

## Conclusion

Potential short-term memory induction constitutes a promising method to enhance physiological responses in SP-CWR. Primed accessions in this study showed physiological mechanisms (delayed senescence, increased foliar area, optimized leaf transpiration, or improved photosynthetic performance) that enable plants to cope with severe drought conditions. Because potential stress memory triggered the greatest increase in foliar area in *I. tiliacea* and some accessions from *I. ramosissima* (CIP 460032, CIP 460722) and *I. triloba* (CIP 460116), this trait might be more relevant in breeding for dual purpose sweetpotato cultivars, used to produce food as well as livestock feed. However, with a view toward long-term memory induction, more studies are required to elucidate the underlying molecular mechanisms (epigenetic processes, gene silencing, chromatin remodeling) responsible for drought tolerance improvement in sweetpotato as shown in this study. Furthermore, we showed that SP-CWR developed drought tolerance through two basic mechanisms: i) resilience, by developing more leaves with an increased time to fix carbon and ii) productivity, by optimizing leaf thermoregulation and gas exchange. The use of resilience capacity and productivity capacity, simultaneously, allowed us to easily identify genotypes from Group C, a group of plants that is highly appreciated by breeders. This study confirms the effectiveness of a potential short-term memory induction for enhancing plants’ drought stress response, the potential applications of which include more efficient water use in irrigated crops and the production of more resilient sweetpotato planting material. It also sets a precedent in stress memory in SP-CWR and demonstrates that this group constitutes a potential and untapped source of valuable physiological traits for sweetpotato improvement programs. However, further field trials under different environmental conditions and in different years are necessary to confirm our preliminary results.

## Data Availability Statement

The original contributions presented in the study are publicly available. This data can be found here: [https://doi.org/10.21223/WNZR93].

## Author Contributions

BH and DR conceived and designed the study. FG-Z gathered the data, analyzed the results, and wrote the manuscript. FG-Z, BH, DR, JR, and JN performed the analysis. FG-Z, BH, DR, JN, JR, and RB edited the manuscript.

## Funding

This investigation was led by North Carolina State University (NCSU), who managed the Sweetpotato CWR project in collaboration with the International Potato Center (CIP) with support from the CGIAR Research Program on Roots, Tubers, and Bananas (RTB). The study was part of the initiative “Adapting Agriculture to Climate Change: Collecting, Protecting and Preparing Crop Wild Relatives” which is supported by the Government of Norway. The initiative is managed by the Global Crop Diversity Trust with the Millennium Seed Bank of the Royal Botanic Gardens, Kew, UK, and implemented in partnership with national and international genebanks and plant breeding institutes around the world. For further information, see Dempewolf et al. (2014), and http://www.cwrdiversity.org/.

## Conflict of Interest

The authors declare that the research was conducted in the absence of any commercial or financial relationships that could be construed as a potential conflict of interest.
