# Supplementary material for: Potential Short-Term Memory Induction as a Promising Method for Increasing Drought Tolerance in Sweetpotato Crop Wild Relatives [Ipomoea series Batatas (Choisy) D. F. Austin]
Source: Front Plant Sci. 2020 Sep 3;11:567507. doi: 10.3389/fpls.2020.567507 (PMC7494806; doi:10.3389/fpls.2020.567507)
Supplement: Supplementary file 1 [file Table_1.docx]

Supplementary Material

Article title: Potential Short-term Memory Induction as a Promising Method for Increasing Drought Tolerance in Sweetpotato Crop Wild Relatives [*Ipomoea* series *Batatas* (Choisy) D. F. Austin]

Authors: Fernando Guerrero-Zurita, David A. Ramírez, Javier Rinza, Johan Ninanya, Raúl Blas and Bettina Heider

The following Supplementary Material is available for this article:

**Table S1:** Environmental conditions during the experiment

**Table S2:** Distribution of accessions according to their flowering status (early, late or absent) and the date (in days after transplanting, DAT) of priming process initiation

**Appendix A:** Calculation of new score indices: resilient capacity index (RCI) and production capacity index (PCI)

**Table S3:** F-values for ANOVA for repeated measurements in time for chlorophyll concentration (Chl_SPAD_), foliar area (FA) and leaf-minus-air temperature (dT) for all 59 accessions used in the study

**Table S4:** Average ± standard error g_s_max_ values for non-primed and primed treatments of 19 accessions

**Table S5:** Scores assigned for each tenth part of the global range of stress susceptibility index (SSI) and geometric mean productivity (GMP) response

**Table S1:** Environmental conditions during the experiment **-** 2018

|  | **September** | **October** | **November** | **December** |
| --- | --- | --- | --- | --- |
| Maximum temperature (ºC) | 34.9 ± 0.26 | 32.3 ± 0.58 | 31.7 ± 0.45 | 32.0 ± 0.54 |
| Minimum temperature (ºC) | 18.6 ± 0.32 | 20.0 ± 0.11 | 20.7 ± 0.19 | 19.8 ± 0.23 |
| Average temperature (ºC) | 24.7 ± 0.19 | 24.2 ± 0.23 | 24.7 ± 0.20 | 24.1 ± 0.25 |
| Maximum relative humidity (%) | 94.2 ± 0.37 | 96.8 ± 0.17 | 96.7 ± 0.24 | 96.0 ± 0.48 |
| Minimum relative humidity (%) | 35.1 ± 1.01 | 49.3 ± 2.03 | 53.1±1.83 | 51.4 ± 2.24 |
| Average relative humidity (%) | 81.2 ± 0.73 | 81.6 ± 0.90 | 81.4 ± 0.91 | 80.8 ± 1.30 |
| Average daily global SR (MJm^−2^day^−1^) | 20.3 ± 0.59 | 17.9 ± 0.94 | 17.9 ± 1.01 | 17.8 ± 1.19 |
| Total precipitation (mm) | 21.4 | 142.6 | 123.8 | 114.6 |
| Average VPD (KPa) | 1.1 ± 0.04 | 0.7 ± 0.05 | 0.7 ± 0.05 | 0.7 ± 0.06 |
| Maximum VPD (KPa) | 3.6 ± 0.09 | 2.5 ± 0.15 | 2.2 ± 0.14 | 2.4 ± 0.1 |

*SR: solar radiation, VPD: vapor pressure deficit.*

**Table S2:** Distribution of accessions according to their flowering status (early, late or absent) and the date (in days after transplanting, DAT) of priming process initiation

| 46 DAT |  | 72 DAT | |
| --- | --- | --- | --- |
| Early |  | Late | Absent |
| CIP 460345 |  | CIP 460360 | CIP 430434 |
| CIP 460585 |  | CIP 460296 | CIP 460577 |
| CIP 460201 |  | CIP 460164 | CIP 440132 |
| CIP 460583 |  | CIP 460077 | CIP 440166 |
| CIP 460204 |  | CIP 460556 | CIP 113641.086 |
| CIP 460026 |  | CIP 460555 | CIP 105269.232 |
| CIP 460430 |  | CIP 460452 | CIP 460149 |
| CIP 460377 |  | CIP 460337 | CIP 460028 |
| CIP 460745 |  | CIP 460610 | CIP 460036 |
| CIP 460663 |  | CIP 460619 | CIP 460032 |
| CIP 460429 |  | CIP 460560 | CIP 460566 |
| CIP 460096 |  | CIP 460309 | CIP 460567 |
| CIP 460195 |  | CIP 460052 | CIP 460005 |
| CIP 460022 |  | CIP 460093 | CIP 460047 |
| CIP 113735.258 |  | CIP 460517 | CIP 460722 |
| CIP 113735.283 |  | CIP 460784 | CIP 460131 |
| CIP 113735.302 |  | CIP 460078 | CIP 460373 |
| CIP 113735.329 |  | CIP 460116 | CIP 460383 |
| CIP 107665.9 |  |  | CIP 460528 |
| CIP 107665.19 |  |  | CIP 460531 |
|  |  |  | CIP 460021 |

**Appendix A:** Calculation of new score indices: resilient capacity index (RCI) and production capacity index (PCI)

Stress susceptibility index (SSI) and geometric mean production (GMP) were transformed into a 1-10 score scale representing RCI and PCI, respectively following the methodology described by Thiry et al. (2016). This score scale is based on the global response per index (SSI and GMP) within the overall population under study (in total 59 accessions). The SSI and GMP values were distributed into ten parts, therefore, each part represents 10%, 20%, ... or 100% of its respective global range of values. For example, for RCI the score value of 9 was assigned to all SSI values between 10 and 20%, while for PCI, the same score value of 9 was assigned to all GMP values between 80 and 90% (**Table S5**). Consequently, low values indicate susceptible stress response (high SSI and low GMP) and high values indicate tolerant stress response (low SSI and high GMP). To assign a score to each index value, a Microsoft Excel tool was developed.

**Table S3:** F-values for repeated measurements ANOVA for chlorophyll concentration (Chl_SPAD_), foliar area (FA) and leaf-minus-air temperature (dT) for all 59 accessions used in this study

|  | **Chl_SPAD_** | **FA** | **dT** |  | **Chl_SPAD_** | **FA** | **dT** |  | **Chl_SPAD_** | **FA** | **dT** |
| --- | --- | --- | --- | --- | --- | --- | --- | --- | --- | --- | --- |
| CIP 460204 |  |  |  | CIP 460619 |  |  |  | CIP 460028 |  |  |  |
| Watering (W) | 4.67 ns | 95.36** | 29.21** | Watering (W) | 9.74* | 7.99* | 48.77** | Watering (W) | 4.41 ns | 75.03** | 3.83 ns |
| Time (T) | 37.93** | 10.65** | 61.95** | Time (T) | 64.70** | 133.01** | 36.28** | Time (T) | 73.52** | 26.69** | 38.41** |
| W × T | 18.19** | 21.02** | 8.18** | W × T | 18.38** | 69.37** | 9.90** | W × T | 5.51** | 10.53** | 7.52* |
| CIP 460345 |  |  |  | CIP 460556 |  |  |  | CIP 460036 |  |  |  |
| Watering (W) | 0.00 ns | 209.90** | 233.63** | Watering (W) | 3.42 ns | 14.27** | 51.40** | Watering (W) | 8.90* | 100.51** | 15.99** |
| Time (T) | 16.00** | 11.24** | 37.73** | Time (T) | 55.21** | 77.18** | 33.43** | Time (T) | 41.47** | 130.28** | 31.95** |
| W × T | 4.78** | 23.68** | 14.98** | W × T | 9.24** | 28.33** | 3.31 ns | W × T | 3.76** | 19.88** | 11.72** |
| CIP 460585 |  |  |  | CIP 460560 |  |  |  | CIP 460032 |  |  |  |
| Watering (W) | 2.23 ns | 188.55** | 72.65** | Watering (W) | 0.64 ns | 29.90** | 8.12* | Watering (W) | 36.50** | 0.04 ns | 1.93 ns |
| Time (T) | 15.89** | 20.14** | 50.94** | Time (T) | 63.42** | 55.50** | 31.75** | Time (T) | 57.38** | 55.72** | 47.99** |
| W × T | 8.99** | 31.38** | 22.71** | W × T | 4.18** | 18.41** | 22.37** | W × T | 14.71** | 27.97** | 2.29 ns |
| CIP 460201 |  |  |  | CIP 460309 |  |  |  | CIP 460566 |  |  |  |
| Watering (W) | 0.27 ns | 112.77** | 135.01** | Watering (W) | 0.07 ns | 45.67** | 8.79* | Watering (W) | 0.00 ns | 36.63** | 8.16* |
| Time (T) | 15.31** | 22.27** | 172.45** | Time (T) | 44.01** | 82.73** | 41.61** | Time (T) | 66.03** | 144.73** | 102.47** |
| W × T | 2.74* | 26.86** | 45.39** | W × T | 4.30** | 21.85** | 2.85 ns | W × T | 22.45** | 29.59** | 3.16 ns |
| CIP 460583 |  |  |  | CIP 460077 |  |  |  | CIP 460567 |  |  |  |
| Watering (W) | 1.75 ns | 72.31** | 63.27** | Watering (W) | 3.59 ns | 56.53** | 8.31* | Watering (W) | 11.60* | 162.70** | 0.52 ns |
| Time (T) | 17.86** | 4.92* | 58.55** | Time (T) | 64.81** | 49.77** | 23.99** | Time (T) | 62.12** | 116.31** | 153.95** |
| W × T | 1.99 ns | 2.16 ns | 6.12* | W × T | 5.01** | 16.75** | 5.25* | W × T | 22.19** | 23.11** | 5.91* |
| CIP 460026 |  |  |  | CIP 460360 |  |  |  | CIP 460005 |  |  |  |
| Watering (W) | 0.99 ns | 78.72** | 221.18** | Watering (W) | 5.53 ns | 7.23* | 17.70** | Watering (W) | 2.59 ns | 554.65** | 16.01** |
| Time (T) | 14.23** | 86.36** | 66.52** | Time (T) | 63.36** | 41.18** | 64.61** | Time (T) | 39.46** | 89.20** | 118.20** |
| W × T | 7.43** | 34.61** | 11.17** | W × T | 8.24** | 10.05** | 8.29* | W × T | 3.11** | 35.70** | 11.60** |
| CIP 460430 |  |  |  | CIP 460052 |  |  |  | CIP 460047 |  |  |  |
| Watering (W) | 0.74 ns | 117.69** | 1299.68** | Watering (W) | 18.03** | 56.85** | 38.70** | Watering (W) | 22.73** | 235.58** | 3.99 ns |
| Time (T) | 10.85** | 32.60** | 70.42** | Time (T) | 74.39** | 78.09** | 31.74** | Time (T) | 20.17** | 59.38** | 166.21** |
| W × T | 8.84** | 16.06** | 25.49** | W × T | 10.86** | 28.21** | 9.23** | W × T | 7.46** | 19.43** | 19.94** |
| CIP 460377 |  |  |  | CIP 460452 |  |  |  | CIP 460722 |  |  |  |
| Watering (W) | 44.08** | 20.50** | 182.78** | Watering (W) | 2.98 ns | 21.35** | 51.12** | Watering (W) | 10.33* | 5.61 ns | 40.20** |
| Time (T) | 6.88** | 125.80** | 85.06** | Time (T) | 111.40** | 29.55** | 21.93** | Time (T) | 44.40** | 29.64** | 247.18** |
| W × T | 1.20 ns | 73.12** | 57.78** | W × T | 7.82** | 15.32** | 1.58 ns | W × T | 11.82** | 13.24** | 8.47** |
| CIP 460745 |  |  |  | CIP 460093 |  |  |  | CIP 430434 |  |  |  |
| Watering (W) | 5.78 ns | 26.37** | 67.88** | Watering (W) | 5.67 ns | 4.13 ns | 13.35* | Watering (W) | 3.73 ns | 99.88** | 2.68 ns |
| Time (T) | 3.59** | 245.86** | 46.34** | Time (T) | 101.46** | 49.62** | 24.65** | Time (T) | 42.50** | 250.26** | 338.56** |
| W × T | 5.65** | 86.59** | 27.04** | W × T | 3.07* | 18.12** | 19.25** | W × T | 9.48** | 72.16** | 9.25** |
| CIP 460663 |  |  |  | CIP 460517 |  |  |  | CIP 460577 |  |  |  |
| Watering (W) | 158.28** | 2.41 ns | 212.18** | Watering (W) | 5.85 ns | 38.24** | 13.90** | Watering (W) | 0.38 ns | 67.27** | 0.91 ns |
| Time (T) | 22.24** | 66.50** | 115.85** | Time (T) | 135.32** | 182.63** | 17.78** | Time (T) | 66.71** | 96.95** | 86.67** |
|  | 13.56** | 32.79** | 59.90** | W × T | 10.88** | 60.95** | 12.02** | W × T | 11.72** | 19.80** | 6.83* |

**TABLE S3:** Continued

|  | **Chl_SPAD_** | **FA** | **dT** |  | **Chl_SPAD_** | **FA** | **dT** |  | **Chl_SPAD_** | **FA** | **dT** |
| --- | --- | --- | --- | --- | --- | --- | --- | --- | --- | --- | --- |
| CIP 460429 |  |  |  | CIP 460784 |  |  |  | CIP 460021 |  |  |  |
| Watering (W) | 0.07 ns | 12.51* | 14.16** | Watering (W) | 25.06** | 35.44** | 12.53* | Watering (W) | 2.32 ns | 4.90 ns | 36.65** |
| Time (T) | 11.60** | 203.75** | 34.96** | Time (T) | 242.01** | 150.16** | 15.48** | Time (T) | 29.34** | 19.71** | 149.60** |
| W × T | 4.24** | 80.63** | 10.33** | W × T | 23.59** | 39.09** | 1.87 ns | W × T | 5.97** | 5.78* | 12.38** |
| CIP 460096 |  |  |  | CIP 460078 |  |  |  | CIP 460528 |  |  |  |
| Watering (W) | 1.41 ns | 47.14** | 27.90** | Watering (W) | 0.92 ns | 32.47** | 9.97* | Watering (W) | 12.64* | 11.72* | 6.05* |
| Time (T) | 4.91** | 109.39** | 35.37** | Time (T) | 82.20** | 64.75** | 8.35** | Time (T) | 48.06** | 69.70** | 147.41** |
| W × T | 7.49** | 36.88** | 5.35* | W × T | 4.79** | 25.54** | 3.68 ns | W × T | 10.82** | 22.49** | 5.72* |
| CIP 460195 |  |  |  | CIP 460116 |  |  |  | CIP 460531 |  |  |  |
| Watering (W) | 4.83 ns | 176.05** | 15.87** | Watering (W) | 80.13** | 7.02* | 90.22** | Watering (W) | 7.83* | 0.31 ns | 15.90** |
| Time (T) | 22.90** | 167.92** | 28.43** | Time (T) | 50.06** | 88.33** | 30.76** | Time (T) | 51.40** | 59.78** | 320.23** |
| W × T | 1.19 ns | 55.80** | 13.84** | W × T | 6.03** | 34.21** | 6.29* | W × T | 10.72** | 22.91** | 6.49* |
| CIP 460022 |  |  |  | CIP 460337 |  |  |  | CIP 460373 |  |  |  |
| Watering (W) | 1.02 ns | 5.35 ns | 78.19** | Watering (W) | 7.68 ns | 78.60** | 14.94* | Watering (W) | 9.67* | 5.67 ns | 141.38** |
| Time (T) | 3.72** | 36.86** | 15.67** | Time (T) | 20.62** | 13.99** | 4.74** | Time (T) | 38.36** | 87.94** | 121.11** |
| W × T | 2.28* | 15.84** | 8.03** | W × T | 9.76** | 31.94** | 9.23** | W × T | 8.29** | 29.35** | 6.01* |
| CIP 113735.258 |  |  |  | CIP 460296 |  |  |  | CIP 460383 |  |  |  |
| Watering (W) | 0.48 ns | 307.46** | 21.55** | Watering (W) | 0.01 ns | 91.34** | 28.86** | Watering (W) | 2.60 ns | 13.68* | 38.11** |
| Time (T) | 15.06** | 106.12** | 131.97** | Time (T) | 87.33** | 142.04** | 56.58** | Time (T) | 42.79** | 113.20** | 146.33** |
| W × T | 2.85* | 33.72** | 28.64** | W × T | 13.80** | 64.23** | 13.29** | W × T | 6.10** | 45.14** | 3.23 ns |
| CIP 113735.283 |  |  |  | CIP 460164 |  |  |  | CIP 440132 |  |  |  |
| Watering (W) | 7.54* | 0.99 ns | 14.05** | Watering (W) | 33.27** | 111.89** | 69.12** | Watering (W) | 0.21 ns | 28.89** | 19.77** |
| Time (T) | 16.02** | 39.08** | 26.00** | Time (T) | 52.54** | 158.35** | 32.32** | Time (T) | 47.53** | 21.76** | 138.98** |
| W × T | 5.40** | 15.51** | 1.90 ns | W × T | 21.18** | 39.78** | 9.57** | W × T | 13.51** | 9.90** | 4.50* |
| CIP 113735.302 |  |  |  | CIP 460131 |  |  |  | CIP 440166 |  |  |  |
| Watering (W) | 0.49 ns | 15.85** | 12.70* | Watering (W) | 1.47 ns | 157.63** | 1.85 ns | Watering (W) | 22.58** | 73.63** | 0.50 ns |
| Time (T) | 10.59** | 78.44** | 12.69** | Time (T) | 62.36** | 351.73** | 66.93** | Time (T) | 23.04** | 39.25** | 94.74** |
| W × T | 4.05** | 31.48** | 5.66* | W × T | 1.31 ns | 57.25** | 8.93** | W × T | 14.73** | 20.64** | 0.98 ns |
| CIP 113735.329 |  |  |  | CIP 460149 |  |  |  | CIP 113641.086 |  |  |  |
| Watering (W) | 22.04** | 216.18** | 0.68 ns | Watering (W) | 0.20 ns | 20.21** | 4.77 ns | Watering (W) | 8.76* | 29.76** | 23.39** |
| Time (T) | 25.32** | 106.79** | 58.93** | Time (T) | 39.05** | 67.53** | 23.72** | Time (T) | 19.10** | 21.54** | 226.64** |
| W × T | 8.34** | 20.60** | 13.73** | W × T | 3.06* | 6.23* | 2.69 ns | W × T | 9.26** | 5.64* | 5.20* |
| CIP 107665.9 |  |  |  | CIP 460555 |  |  |  | CIP 105269.232 |  |  |  |
| Watering (W) | 4.91 ns | 0.27 ns | 61.76** | Watering (W) | 3.47 ns | 4.82 ns | 4.82 ns | Watering (W) | 2.73 ns | 24.43** | 38.45** |
| Time (T) | 26.82** | 43.68** | 216.51** | Time (T) | 90.81** | 61.62** | 6.87** | Time (T) | 56.54** | 49.75** | 189.15** |
| W × T | 13.12** | 22.17** | 48.46** | W × T | 22.10** | 20.95** | 2.45 ns | W × T | 8.31** | 45.85** | 3.35 ns |
| CIP 107665.19 |  |  |  | CIP 460610 |  |  |  |  |  |  |  |
| Watering (W) | 15.78** | 13.59* | 54.05** | Watering (W) | 0.25 ns | 45.84** | 17.48** |  |  |  |  |
| Time (T) | 20.66** | 29.27** | 29.67** | Time (T) | 55.44** | 232.14** | 87.44** |  |  |  |  |
| W × T | 2.69* | 10.74* | 10.95** | W × T | 5.76** | 13.17** | 5.93* |  |  |  |  |

**Table S4:** Average ± standard error g_s_max_ values for non-primed and primed treatments of 19 accessions

|  | g_s_max_ (mol H_2_O m^-2^ s^-1^) | |
| --- | --- | --- |
| Accessions | Non-primed | Primed |
| CIP 107665.19 | 0.121 ± 0.10 | 0.116 ± 0.05 |
| CIP 460022 | 0.131 ± 0.06 | 0.072 ± 0.06 |
| CIP 113735.258 | 0.127 ± 0.03 | 0.052 ± 0.01 |
| CIP 460201 | 0.045 ± 0.01 | 0.017 ± 0.00 |
| CIP 113735.283 | 0.166 ± 0.07 | 0.036 ± 0.02 |
| CIP 460026 | 0.097 ± 0.03 | 0.020 ± 0.01 |
| CIP 107665.9 | 0.055 ± 0.04 | 0.010 ± 0.01 |
| CIP 460585 | 0.057 ± 0.03 | 0.008 ± 0.00 |
| CIP 113735.302 | 0.095 ± 0.04 | 0.013 ± 0.00 |
| CIP 460583 | 0.082 ± 0.05 | 0.010 ± 0.01 |
| CIP 460195 | 0.101 ± 0.02 | 0.011 ± 0.01 |
| CIP 460096 | 0.103 ± 0.02 | 0.009 ± 0.01 |
| CIP 460345 | 0.066 ± 0.02 | 0.005 ± 0.00 |
| CIP 460430 | 0.076 ± 0.01 | 0.006 ± 0.00 |
| CIP 460745 | 0.165 ± 0.05 | 0.010 ± 0.02 |
| CIP 460663 | 0.184 ± 0.01 | 0.010 ± 0.02 |
| CIP 460429 | 0.103 ± 0.04 | 0.005 ± 0.01 |
| CIP 460204 | 0.056 ± 0.01 | 0.002 ± 0.00 |
| CIP 460377 | 0.118 ± 0.08 | 0.004 ± 0.00 |

**Table S5:** Scores assigned for each tenth part of the global range of stress susceptibility index (SSI) and geometric mean productivity (GMP) response

| Scores | SSI range | GMP range |
| --- | --- | --- |
| 1 | [1.74 – 1.48˃ | [7.6 – 14.6˃ |
| 2 | [1.48 – 1.23˃ | [14.6 – 21.7˃ |
| 3 | [1.23 – 0.97˃ | [21.7 – 28.7˃ |
| 4 | [0.97 – 0.71˃ | [28.7 – 35.8˃ |
| 5 | [0.71 – 0.45˃ | [35.8 – 46.6˃ |
| 6 | [0.45 – 0.20˃ | [46.6 – 49.9˃ |
| 7 | [0.20 – -0.06˃ | [49.9 – 57.0˃ |
| 8 | [-0.06 – -0.32˃ | [57.0 – 64.0˃ |
| 9 | [-0.32 – -0.58˃ | [64.0 – 71.1˃ |
| 10 | [-0.58 – -0.83] | [71.1 – 78.1] |
